# Cedarwood Oils: The Wood Essential Oil Compositions from Trees Known as “Cedar”

**DOI:** 10.3390/plants15040659

**Published:** 2026-02-21

**Authors:** William N. Setzer, Prabodh Satyal

**Affiliations:** Aromatic Plant Research Center, 230 N 1200 E, Suite 100, Lehi, UT 84043, USA; psatyal@aromaticplant.org

**Keywords:** *Callitropsis*, *Calocedrus*, *Cedrela*, *Cedrus*, *Chamaecyparis*, *Cryptomeria*, *Cupressus*, *Juniperus*, *Thuja*, *Widdringtonia*

## Abstract

In addition to the true cedars (*Cedrus* species), there are several genera of trees commonly called “cedar”, including species of *Callitropsis*, *Calocedrus*, *Cedrela*, *Chamaecyparis*, *Cryptomeria*, *Cupressus*, *Juniperus*, *Thuja*, and *Widdringtonia*. The wood essential oils (cedarwood oils) of these trees have been used as flavor and fragrance materials, as well as in medicinal applications. In this study, we present summaries of the wood essential oils from trees known as “cedar”. A literature search was carried out on cedarwood oils and, when available, compared with commercial wood essential oils from the Aromatic Plant Research Center (APRC) collection. *Cedrus* wood oils are generally dominated by the himachalenes and atlantones. Sesquiterpenoids are abundant in other cedarwood oils, including cedrenes, cedrol, and thujopsene in *Cupressus funebris*, *Juniperus ashei*, and *Juniperus virginiana*. Cadinane sesquiterpenoids are generally abundant in *Cedrela odorata* and *Cryptomeria japonica*, while nootkatane sesquiterpenoids are found in *Callitropsis nootkatensis* and eudesmane sesquiterpenoids are found in *Thuja occidentalis*. Sesquiterpenoids are generally responsible for the woody fragrances of cedarwood oils, but monoterpenoids can also be dominant (e.g., *Calocedrus* species).

## 1. Introduction

Essential oils, the volatile components of plants, can be derived from different plant tissues, including leaves, e.g., tea tree (*Melaleuca alternifolia* (Maiden & Betche) Cheel) oil, rosemary (*Salvia rosmarinus* Spenn.) oil, and eucalyptus (*Eucalyptus globulus* Labill.) oil; flowers, e.g., rose (*Rosa damascena* Mill.) oil, clove (*Syzygium aromaticum* (L.) Merr. & L.M. Perry) oil, and ylang-ylang (*Cananga odorata* (Lam.) Hook.f. & Thomson) oil; fruits, e.g., juniper (*Juniperus communis* L.) oil; fruit rinds, e.g., bergamot (*Citrus bergamia* (Risso) Risso & Poit.) oil and lemon (*Citrus limon* (L.) Osbeck) oil; seeds, e.g., anise (*Illicium verum* Hook.f.) oil and caraway (*Carum carvi* L.) oil; roots, e.g., calamus (*Acorus calamus* L.) oil, ginger (*Zingiber officinale* Roscoe) oil, and valerian (*Valeriana jatamansi* Jones) oil; bark, e.g., cinnamon (*Cinnamomum verum* J. Presl) oil and sweet birch (*Betula lenta* L.) oil; or resin, e.g., copaiba (*Copaifera epunctata* Amshoff) oil, frankincense (*Boswellia carteri* Birdw.) oil, and myrrh (*Commiphora myrrha* (T. Nees) Engl.) oil. However, the heartwood of trees can also be a source of essential oils. Some of the more common wood essential oils are listed below.

Rosewood essential oil is steam-distilled from the wood of *Aniba rosaeodora* Ducke or *Aniba duckei* Kosterm. and is a valuable essential oil in perfumery, as well as folk medicine [1] and aromatherapy [2]. There has been some debate in the botanical literature as to whether *A. rosaeodora* and *A. duckei* (syn. *A. rosaeodora* var. *amazonica* Ducke) are separate species [3]. Both species occur in the Amazon region of South America, ranging along the Amazon drainage basin from Loreto, Peru, east to Amapá, Brazil. Rosewood oil is dominated by the monoterpenoid alcohol linalool (75–99%) [4,5], and that compound accounts for its value as a perfume ingredient, as well as its physiological properties. Rosewood oil has been shown to reduce anxiety [2], induce sedation [6] and reduce blood pressure and heart rate [1]. However, because of over-harvesting, the tree is endangered, and alternatives to rosewood oil have been emerging [3].

Sandalwood essential oil is obtained by steam distillation of the heartwood of East Indian sandalwood, *Santalum album* L. This tree is a root parasite that burrows its roots into nearby trees during the early part of its development (around seven years). Heartwood formation in *S. album* occurs only after about 30 years, so generally only trees > 60 cm diameter are harvested [7]. The use of sandalwood is documented in Sanskrit manuscripts more than 4000 years old. The oil is used extensively in perfumery, as well as medicinally [7]. The most important constituents of East Indian sandalwood oil are α-santalol (40–47%), β-santalol (18–21%), and *trans*-α-bergamotol (6–9%) [8,9]. α-Santalol has been described as having a slightly woody odor, while β-santalol is more potent with woody, musky, animal qualities [10]. Because of over-harvesting, cheaper alternative sources for East Indian sandalwood oil have been emerging, as well as adulteration of “sandalwood oil”. Alternatives to East Indian sandalwood oil include *Santalum spicatum* (R.Br.) A.DC. from West Australia, *Santalum austrocaledonicum* Vieill. from New Caledonia [8], and *Santalum paniculatum* Hook. & Arn. from Hawaii [11]. *S. spicatum* oil is not considered an appropriate substitute for *S. album* oil [8]; *S. spicatum* oil only contains 10–22% α-santalol and 4–8% β-santalol [12,13].

Cedarwood essential oils are typically produced by steam-distilling the woods of several different species of junipers (*Juniperus* spp.), cypresses (*Cupressus* spp.), or true cedars (*Cedrus* spp.). The true cedars are members of the *Cedrus* genus (Pinaceae), although several other genera (e.g., *Juniperus*, *Cupressus*, and *Chamaecyparis*) include species that are referred to as “cedar” in common nomenclature. The purpose of this review is to examine the essential oil compositions of wood essential oils from trees commonly called “cedar” (Table 1).

**Figure 1:**
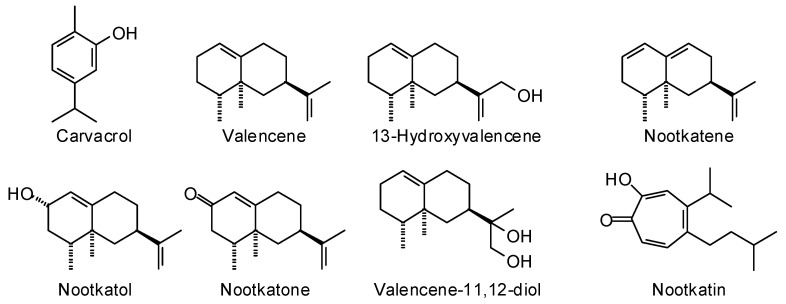
Major components in the wood essential oil of *Callitropsis nootkatensis*.

*Callitropsis nootkatensis* (D. Don) Oerst. (Cupressaceae) is native to northwestern North America, from coastal Alaska, south through coastal British Columbia, through the Cascades of Washington and Oregon, and barely reaching northern California [14]. Three essential oil samples have been reported, and two commercial essential oils are reported in Table 2. The chemical structures are shown in Figure 1. Based on the limited number of samples, there are apparently two chemotypes based on the wood essential oil compositions, a carvacrol-rich chemotype and a nootkatene-rich chemotype. The heartwood essential oil of *C. nootkatensis* (rich in nootkatone, valencene-11,12-diol, carvacrol, and nootkatol) has shown antifungal activity against the plant pathogenic fungus *Phytophthora ramorum* (strongly inhibitory at a concentration of 10,000 mg/kg) [15]. In addition, nootkatin and valencene-11,12-diol, isolated from *C. nootkatensis*, showed strong (EC_50_ 10 ppm) and moderate (EC_50_ 66 ppm) inhibition, respectively, of *P. ramorum* [16]. The major components of *C. nootkatensis* wood essential oil (carvacrol, valencene, nootkatene, nootkatone, valencen-13-ol, and nootkatol) have shown notable pesticidal activity against the rat flea (*Xenopsylla cheopis*), the deer tick (*Ixodes scapularis*), and the yellow fever mosquito (*Aedes aegypti*) [17]. For example, the major component nootkatene showed 24-h LC_50_ values of 170, 110, and 270 ppm, respectively.

**Figure 2:**
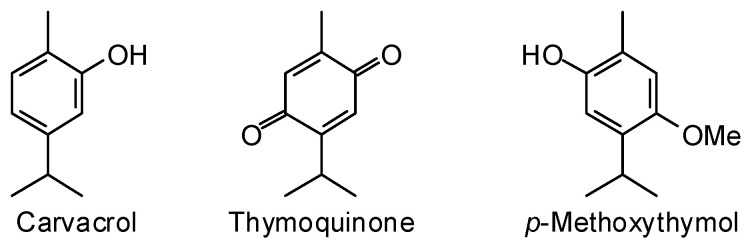
Major components in the wood essential oil of *Calocedrus decurrens*.

*Calocedrus decurrens* (Torr.) Florin (Cupressaceae), the California incense cedar, is native to the montane forests from Oregon through California and into Baja California, Mexico [14]. The heartwood essential oil of *C. decurrens* has been analyzed and showed thymoquinone (35.9%), carvacrol (29.2%), and *p*-methoxythymol (11.0%) to be the major components [21] (Figure 2). The heartwood essential oil (composition not reported) has shown biocidal activities to nymphal ticks (*Ixodes scapularis*) (24-h LC_50_ = 96 ppm), adult fleas (*Xenopsylla cheopis*) (24-h LC_50_ = 240 ppm), and adult mosquitoes (*Aedes aegypti*) (24-h LC_50_ = 5 ppm) [22]. Carvacrol showed pesticidal activities with 24-h LC_50_ values of 68, 59, and 51 ppm against those organisms, respectively [17].

**Figure 3:**
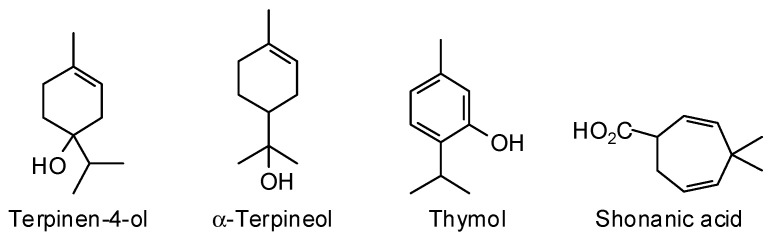
Major components in the wood essential oil of *Calocedrus formosana*.

*Calocedrus formosana* (Florin) Florin (Cupressaceae) is native to the mountains of northern Taiwan [23]. The major components in the wood essential oil of *C. formosana* were α-terpineol (23.5%), terpinen-4-ol (12.2%), shonanic acid (10.5%), and thymol (5.3%) [24] (Figure 3). The wood essential oil (composition not reported) showed anti-termitic activity against *Coptotermes formosanus* with a 24-h LC_50_ of 2600 ppm [25].

**Figure 4:**
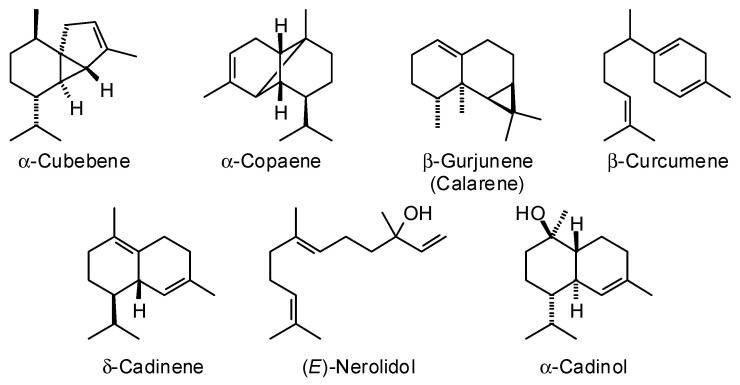
Major components in the wood essential oil of *Cedrela odorata*.

*Cedrela odorata* L. (Meliaceae), Spanish cedar, is native to the Neotropics (Mexico, through Central America, into South America, including the West Indies [26]. There have been a few reports on the wood essential oil of *C. odorata* from different geographical locations (Table 3). The essential oils are dominated by sesquiterpenoids (Figure 4), but there is clearly variation in the essential oil compositions based on geographical location of the source trees.

**Figure 5:**
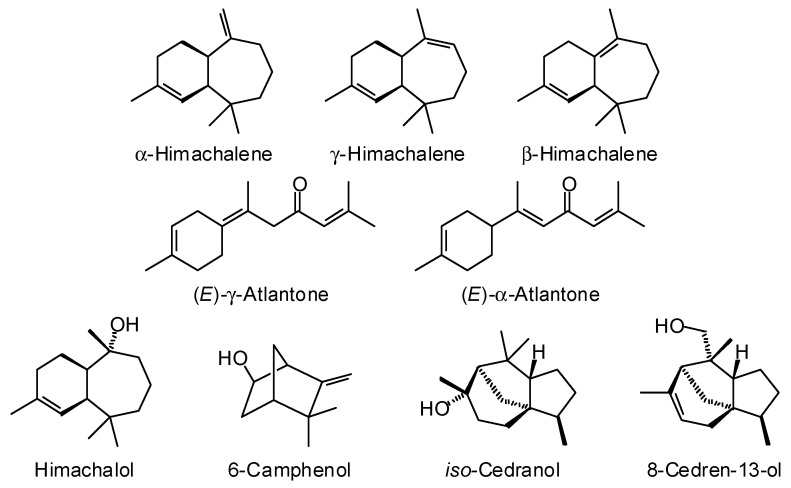
Major components in the wood essential oil of *Cedrus atlantica*.

*Cedrus atlantica* (Endl.) Manetti ex Carrière (Pinaceae), the Atlas cedar, is native to the Rif and Atlas Mountains of North Africa, especially Algeria and Morocco [30]. A total of 48 commercial essential oil samples from the collection of the Aromatic Plant Research Center (APRC) were used to evaluate the major components. The major components in the commercial wood essential oil components of *C. atlantica* are α-himachalene (15.3 ± 1.8%), γ-himachalene (9.9 ± 0.9%), β-himachalene (40.3 ± 4.9%), (*E*)-γ-atlantone (1.9 ± 2.0%), and (*E*)-α-atlantone (5.4 ± 3.4%) (Figure 5). Several published reports on *C. atlantica* wood essential oils mirror these compositional profiles, but many do not (see Table 4). Atlas cedar wood essential oil has shown antibacterial activity [31,32,33,34] (e.g., MIC of 400 ppm against *Escherichia coli* and 200 ppm against *Bacillus subtilis* [34]), weak antifungal activity against wood rot fungi *Gloeophyllum trabeum* (MIC = 1000 ppm), *Oligoporus placenta* (MIC = 2500 ppm), *Coniophora puteana* (MIC = 2500 ppm), and *Trametes versicolor* (MIC = 1250 ppm) [35]; anti-inflammatory and analgesic effects in rodent models [36,37], and in vitro cytotoxic activity on A375 human melanoma and HT-29 colorectal carcinoma cell lines [38].

**Figure 6:**
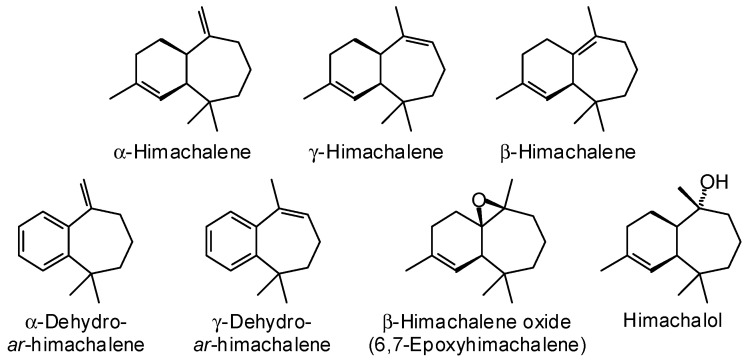
Major components in the wood essential oil of *Cedrus brevifolia*.

*Cedrus brevifolia* (Hook.f.) Elwes & A. Henry (Pinaceae) is endemic to the island of Cyprus (eastern Mediterranean) [48]. Apparently, only one study on the wood essential oil has been published [49]; the major components in the heartwood essential were α-himachalene (17.3%), γ-himachalene (15.0%), β-himachalene (22.0%), α-dehydro-*ar*-himachalene (5.2%), γ-dehydro-*ar*-himachalene (2.0%), β-himachalene oxide (7.0%), and himachalol (10.5%) (Figure 6).

**Figure 7:**
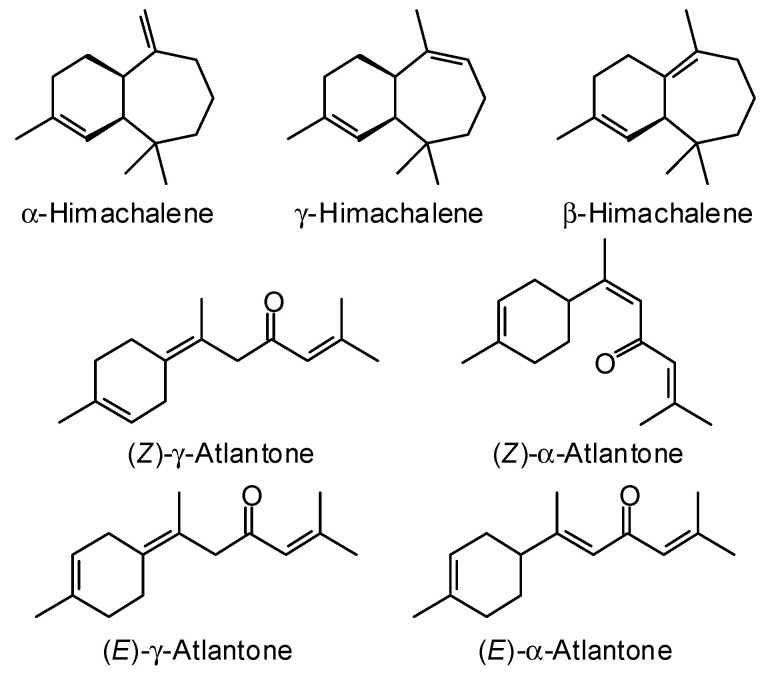
Major components in the wood essential oil of *Cedrus deodara*.

*Cedrus deodara* (Roxb. ex D. Don) G. Don (Pinaceae), Deodar cedar, is native to the western Himalayas (eastern Afghanistan, northern and northwestern Pakistan, north and central India, southwestern Tibet, and western Nepal) [50]. Several reviews of the phytochemistry and pharmacology of *C. deodara* have been published [50,51,52,53,54,55,56,57,58]. A compilation of 43 wood essential oil samples from commercial sources in the APRC collection shows compositions to be similar to those of *C. atlantica* with the major components α-himachalene, γ-himachalene, β-himachalene, (*Z*)-γ-atlantone, (*E*)-γ-atlantone, (*Z*)-α-atlantone, and (*E*)-α-atlantone (Table 5, Figure 7). Two published reports on the wood essential oils of *C. deodara* are in close agreement [59,60]. The wood essential oil of *C. deodara* has shown in vivo analgesic and anti-inflammatory (rodent model), in vivo antispasmodic (feline model, attributed to himachalol), molluscicidal (100% lethality against *Lymnaea auricularia* at 20 ppm), and insecticidal activities (*Callosobruchus analis* and *Musca domestica*, attributed to himachalol and β-himachalene) [50,55].

*Cedrus libani* (L.) A. Rich. (Pinaceae). The natural range of *C. libani* is southern Türkiye and the coastal Mediterranean in Syria and Lebanon [61]. Some reviews have appeared on the chemical composition and biological activities of *C. libani* [61,62,63], and there have been several reports on the chemical compositions of *C. libani* collected from Türkiye and from Lebanon (Table 6). The major components of *C. libani* wood essential oil are α-himachalene (7.1–12.8%), γ-himachalene (4.4–9.1%), β-himachalene (8.1–38.2%), himachalol (1.2–43.1%), and (*E*)-α-atlantone (0.8–19.7%). The structures are depicted in Figure 6 and Figure 7. The wood essential oil showed antibacterial activity (zone-of-inhibition assay) against the Gram-positive organisms *Staphylococcus aureus* and *Enterococcus faecalis* (but was inactive against Gram-negative *Escherichia coli* and *Pseudomonas aeruginosa*), antifungal activity against *Candida albicans*; cytotoxic activity against A375 (human melanoma, IC_50_ = 20.2 ppm) and MDA-MB-231 (human breast adenocarcinoma, IC_50_ = 54.1 ppm) cells [64], and antiviral activity against the herpes simplex virus type 1 (IC_50_ = 440 ppm) [65].

**Figure 8:**
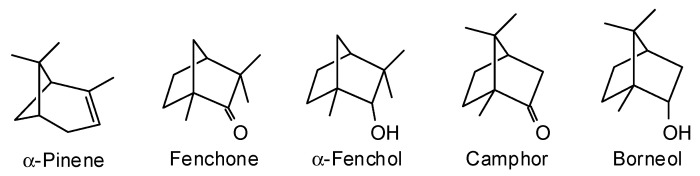

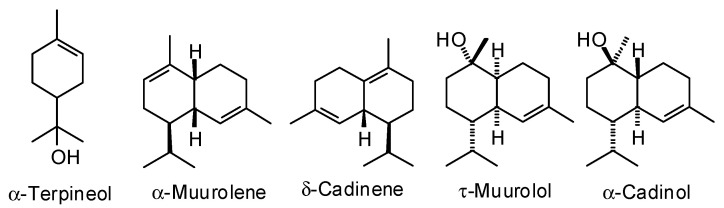
Major volatile components reported in the wood of *Chamaecyparis lawsoniana*.

*Chamaecyparis lawsoniana* (A. Murray bis) Parl. (Cupressaceae) is found naturally growing in an area of coastal Oregon into northern California [67]. There are three reports on the wood volatiles of *C. lawsoniana* [16,68,69] (Table 7, Figure 8). Clearly, there are not enough data available to adequately describe the wood essential oil of *C. lawsoniana*; additional research on this material is necessary.

**Figure 9:**
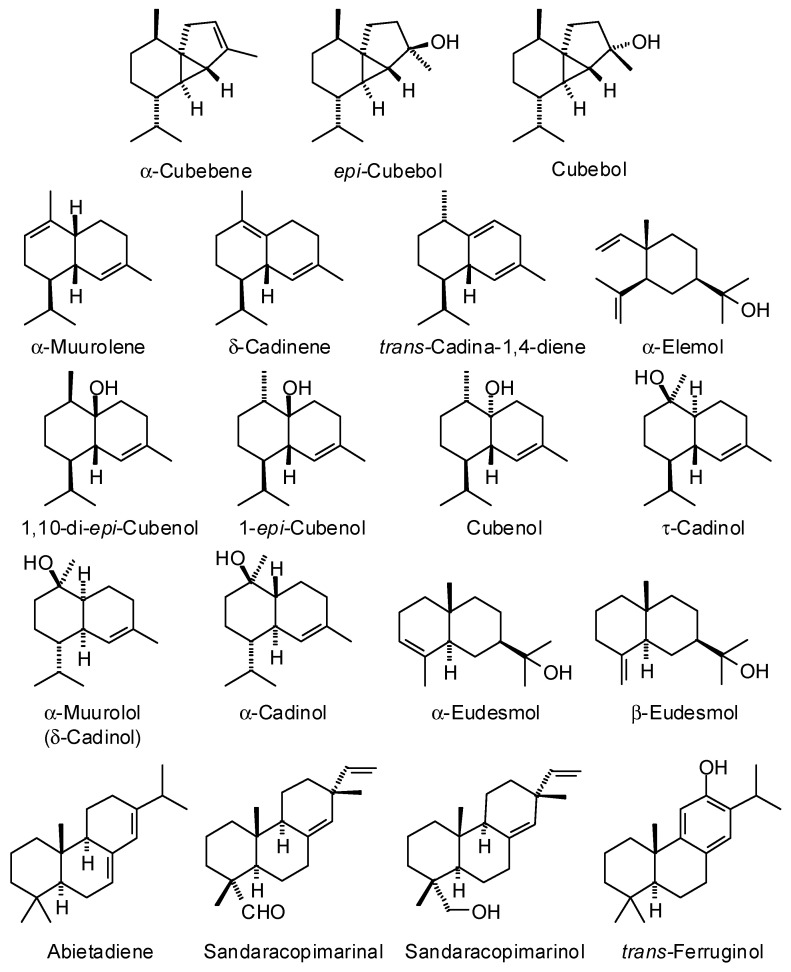
Major components in the wood essential oil of *Cryptomeria japonica*.

*Cryptomeria japonica* (Thunb. ex L.) D. Don (Cupressaceae), the Japanese cedar, is native to Japan [70] but is extensively cultivated in plantations in China and the Azores [71]. The wood essential oils from *C. japonica* have been obtained from Japan, as well as from the Azores (Table 8). Although there are notable quantitative differences in the compositions, the major components are generally *epi*-cubebol, cubebol, δ-cadinene, and β+α-eudesmol. The major components of the wood essential oil are shown in Figure 9. Japanese cedar wood essential oil has demonstrated antifungal activity against *Laetiporus sulphureus* (IC_50_ = 39 ppm), *Collectotrichum gloeosporioides* (IC_50_ = 80 ppm), *Ganoderma australe* (IC_50_ = 110 ppm), *Rhizoctonia solani* (IC_50_ = 65 ppm) [72], *Trichophyton rubrum* (MIC = 313 ppm) [73], and *Aspergillus fumigatus* (MIC, 312–1250 ppm) [74], and has been suggested to maintain mental health in women involved in monotonous work [75,76].

**Figure 10:**
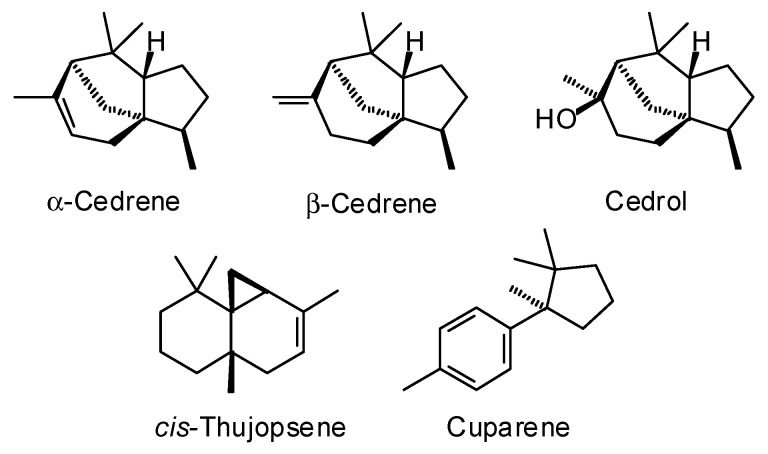
Major components in the wood essential oil of *Cupressus funebris*.

*Cupressus funebris* Endl. (Cupressaceae) predominantly grows in southern China, especially eastern Sichuan, Chongqing, Guizhou, Hunan, Jiangxi, and western Hubei provinces [80]. The wood volatiles from *C. funebris* have shown wide variation, but the major components reported are α-cedrene, β-cedrene, *cis*-thujopsene, cuparene, and cedrol (Table 9, Figure 10). Adams and Li have pointed out that there are problems with the taxonomic identification of *C. funebris* and that commercial “Chinese cedar oil” may be contaminated with wood from other members of the Cupressaceae [81]. Commercial *C. funebris* wood oil has demonstrated tick repellent activity (*Amblyomma americanum*, EC_95_ = 0.465 mg/cm^2^ on filter paper) and mosquito larvicidal activity (*Aedes aegypti*, LC_50_ = 263 ppm) [82].

*Juniperus ashei* J. Buchholz (Cupressaceae). Central Texas is the main locus of *J. ashei*, but there are also populations in northern Mexico, Oklahoma, and Arkansas [84]. The main components in the wood essential oil of *J. ashei* are α-cedrene, β-cedrene, *cis*-thujopsene, and cedrol (Table 10, Figure 10). Texas cedar wood oil was screened for antibacterial activity [85] and for wound-healing activity [86], but was inactive.

**Figure 11:**
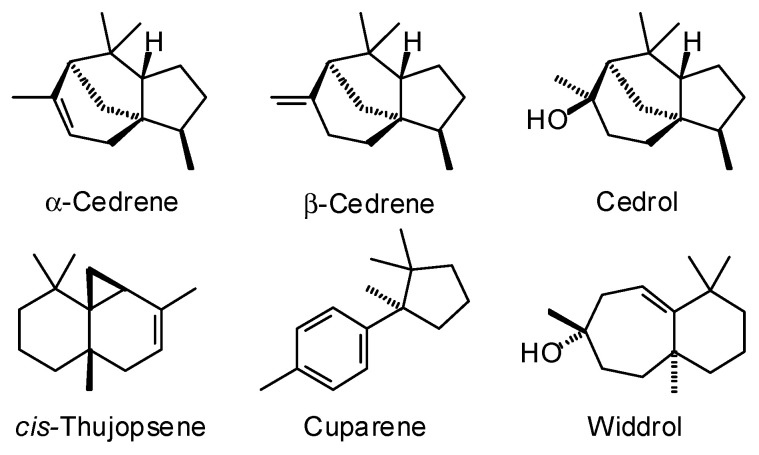
Major components in the wood essential oil of *Juniperus virginiana*.

*Juniperus virginiana* L. (Cupressaceae). The natural range of eastern red cedar is the eastern United States from Michigan, south to Florida, and west to Oklahoma and Kansas [87,88]. A total of 56 commercial wood essential oils from *J. virginiana* have been analyzed at the Aromatic Plant Research Center (APRC). The major components based on the analyses are α-cedrene (31.8 ± 3.8%), β-cedrene (5.8 ± 0.6%), *cis*-thujopsene (19.4 ± 1.5%), widdrol (11.1 ± 2.5%), and cedrol (13.4 ± 2.2%) (Figure 11). Zhang and Rao analyzed a commercial *J. virginiana* sample from France and found a comparable composition with α-cedrene (28.1%), β-cedrene (7.8%), *cis*-thujopsene (17.7%), and cedrol (24.6%), but widdrol was not detected [89]. Adams analyzed *J. virginiana* wood essential oil and found it to have a composition of α-cedrene (27.2%), β-cedrene (7.7%), thujopsene (27.6%), and cedrol (15.8%) [83]. Widdrol was relatively low (1.0%), but cuparene was a major component (6.3%). The commercial samples from APRC showed cuparene to be relatively minor (1.2 ± 0.3%). The wood essential oil of *J. virginiana* has exhibited in vivo anti-inflammatory and wound-healing activities (rodent model) [86]. The wood essential oil has also shown in vivo anxiolytic activity (rodent model) [89], which has been attributed to the major constituent cedrol [90]. Cedrol has demonstrated a number of biological activities including anti-inflammatory, anti-obesity, antifungal, cytotoxic, antimelanogenic, analgesic, and neuroprotective activities, as well as alleviating cerebral ischemia, promoting hair growth [91], and alleviating age-induced cognitive impairment [92].

**Figure 12:**
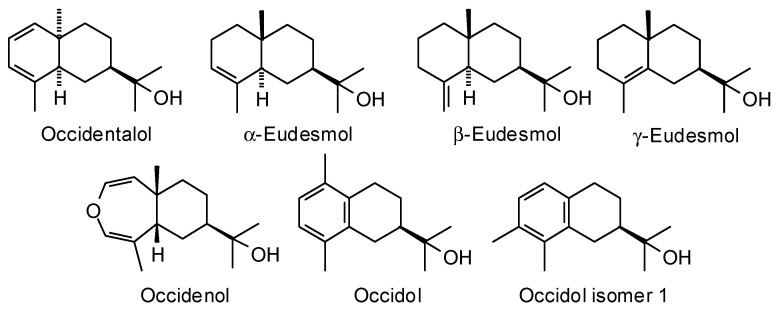
Major components in the wood essential oil of *Thuja occidentalis*.

*Thuja occidentalis* L. (Cupressaceae) ranges broadly across southeastern Canada and northeastern United States, but there is a disjunct range extending south into Ohio, Pennsylvania, Virginia, West Virginia, Kentucky, and Tennessee [93]. The phytochemical and pharmacological properties of the foliage of *T. occidentalis* have been reviewed, but the wood essential oils were not included in the reviews [94,95,96]. However, the wood essential oil of *T. occidentalis* has been obtained and analyzed by Weyerstahl et al. [97] and by Andersen et al. [98] (Table 11, Figure 12).

*Thuja plicata* Donn ex D. Don (Cupressaceae). The major components of *T. plicata* wood essential oil, based on seven samples of commercial essential oil in the APRC collection, are terpinen-4-ol (3.8 ± 2.0%), α-terpineol (2.5 ± 0.8%), methyl myrtenate (4.2 ± 1.2%), and methyl thujate (54.1 ± 7.0%). There have been two reports on the volatiles from *T. plicata* wood. Manter and co-workers extracted the heartwood of *T. plicata* with ethyl acetate and analyzed the extract by GC-MS and found thujic acid (39.9%), nezukone (16.7%), and hinokitiol (6.7%) to be the major volatile components in the extract [16]. Mellouk et al. carried out a steam distillation of *T. plicata* wood, but reported plicatic acid (a polyphenolic lignan, C_18_H_20_O_9_, MW 380.35) to be the major component [99]. This compound is unlikely to be volatile enough to be detected by GC-MS.

**Figure 13:**
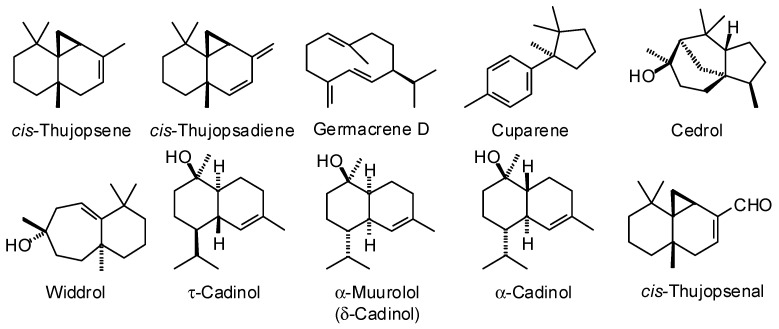
Major components in the wood essential oil of *Widdringtonia* species.

*Widdringtonia* Endl. Species. There are four *Widdringtonia* species, all endemic to southern Africa, *Widdringtonia nodiflora* (L.) Powrie (mountain cedar), *Widdringtonia schwarzii* (Marloth) Mast. (Willowmore cedar), *Widdringtonia wallichii* Endl. (syn. *Widdringtonia cedarbergensis* J.A. Marsh, Clanwilliam cedar), and *Widdringtonia whytei* Rendle (Mulanje cypress) [100]. The major wood essential oil components of *W. nodiflora*, *W. schwartzii*, and *W. whytei* are summarized in Table 12 and Figure 13. The wood essential oil of *W. nodiflora* showed acaricidal activity against several species of ticks, with LC_50_ values in the range of 15.5–39.8 ppm [100].

## 2. Materials and Methods

### 2.1. Literature Search

A literature search was carried out using several scientific databases, including PubMed/MEDLINE, ScienceDirect, Scopus, Web of Science, and Google Scholar. Search terms included the following keywords: *Callitropsis nootkatensis*, *Chamaecyparis nootkatensis*, *Xanthocyparis nootkatensis*, *Calocedrus decurrens*, *Calocedrus formosana*, *Calocedrus macrolepis*, *Cedrela odorata*, *Cedrus atlantica*, *Cedrus brevifolia*, *Cedrus deodara*, *Cedrus libani*, *Chamaecyparis lawsoniana*, *Cryptomeria japonica*, *Cupressus funebris*, *Juniperus ashei*, *Juniperus procera*, *Juniperus virginiana*, *Thuja occidentalis*, *Thuja plicata*, *Widdringtonia*, cedarwood, essential oil. Plant taxonomy was validated using the World Flora Online database [103]. Chemical structures were verified using the Dictionary of Natural Products [104], PubChem [105], and NIST Chemistry WebBook [106].

### 2.2. Essential Oil Analysis

Commercial essential oil samples from the Aromatic Plant Research Center (APRC) collection were analyzed by gas chromatography–mass spectrometry using a Shimadzu GC-MS-QP2010 Ultra (Shimadzu Scientific Instruments, Columbia, MD, USA), equipped with a Zebron ZB-5ms fused silica capillary column (60 m × 0.25 mm × 0.25 μm film thickness) (Phenomenex, Torrance, CA, USA), helium carrier gas, 2.0 mL/min flow rate, injection and ion source temperatures 260 °C; GC oven program 50 °C to 260 °C at 2.0 °C/min; 0.1 μL of a 5% (*w*/*v*) sample of essential oil in dichloromethane injected, split mode, 24.5:1 split ratio.

## 3. Conclusions

This report summarizes the cedarwood essential oil compositions of several different “cedar” species. Interestingly, there are notable differences in the wood essential oils of the “cedar” trees. The true cedars (*Cedrus* species, Pinaceae) are dominated by himachalane and bisabolane sesquiterpenoids. There are subtle differences, however, between *C. atlantica* and *C. deodara*. In *C. atlantica*, the atlantones are typically very low, whereas in *C. deodara*, atlantones usually account for at least 20% of the total composition. *Cedrus deodara* generally contains < 40% β-himachalene, while *C. atlantica* typically exceeds 40%. There are subtle differences in the aroma profiles of *C. atlantica* and *C. deodara*, but they are very similar. Because of the higher price and rarity of *C. atlantica*, it is frequently adulterated with *C. deodara*. *Cupressus*, *Juniperus*, and *Widdringtonia* species (Cupressaceae) are rich in cedrane and thujopsane sesquiterpenoids. Other genera from the Cupressaceae have different essential oil profiles: *Callitropsis* (nootkatane sesquiterpenoids), *Calocedrus* (menthane monoterpenoids), *Chamaecyparis* (camphane monoterpenoids and cadinane sesquiterpenoids), and *Cryptomeria* (cadinane and eudesmane sesquiterpenoids). *Cedrela* (Meliaceae, not a gymnosperm) is rich in cadinane sesquiterpenoids. The information provided in this review should be valuable to companies interested in using cedarwood oils as flavor or fragrance materials; in the fine fragrance and aroma chemical industry (highest total volume), as an industrial fragrance and derivatives, personal care, household/insect control, and in aromatherapy (smallest volume, but high value). In addition, the compositions may help to avoid erroneous analyses, select ideal geographical sources, and avoid cedarwood oils adulterated or contaminated with other tree species. However, there are several “cedar” species that require additional research in order to more confidently describe the wood essential oil compositions. There are serious gaps in research regarding the essential oil characterizations of *Calocedrus decurrens* (only one analysis reported), *Calocedrus formosana* (only one analysis reported), *Cedrela odorata* (only four reliable samples reported), *Cedrus brevifolia* (only one sample reported), *Chamaecyparis lawsoniana* (only one essential oil report), *Thuja occidentalis* (only two samples reported from the 1990s), and *Widdringtonia* species (only one sample each of four species reported). It is important to point out that solvent extracts, including supercritical CO_2_ extracts, are not essential oils. Cedarwood essential oils, in this sense, are obtained by steam distillation or hydrodistillation, thus indicating the need for additional essential oil analyses. As additional essential oil compositional data are obtained, different chemotypes can be defined, “standard” compositions (analogous to ISO standards) can be described, and anomalous components can be identified and perhaps categorized as contaminant or adulterant marker compounds. There is an opportunity for additional screening for the biological activity of cedarwood oils, which may reveal new and important agricultural and medicinal uses.

## Figures and Tables

**Table 1:** The “cedar” trees covered in this review.

Tree Species	Common Name
*Callitropsis nootkatensis* (D. Don) Oerst. (syn. *Chamaecyparis nootkatensis* (D. Don) Spach, *Xanthocyparis nootkatensis* (D. Don) Farjon & D.K. Harder)	Alaska cedar
*Calocedrus decurrens* (Torr.) Florin	California incense cedar
*Calocedrus formosana* (Florin) Florin (syn. *Calocedrus macrolepis* var. *formosana* (Florin) W.C. Cheng & L.K. Fu)	Taiwan incense cedar
*Cedrela odorata* L.	Spanish cedar
*Cedrus atlantica* (Endl.) Manetti ex Carrière	Atlas cedar
*Cedrus brevifolia* (Hook.f.) Elwes & A. Henry (syn. *Cedrus libani* var. *brevifolia* Hook.f.)	Cyprian cedar
*Cedrus deodara* (Roxb. ex D. Don) G. Don	Deodara cedar
*Cedrus libani* (L.) A. Rich.	Cedar of Lebanon
*Chamaecyparis lawsoniana* (A. Murray bis) Parl.	Port Orford cedar
*Cryptomeria japonica* (Thunb. ex L.) D. Don	Japanese cedar
*Cupressus funebris* Endl.	Chinese cedar
*Juniperus ashei* J. Buchholz	Texas cedar
*Juniperus virginiana* L.	Eastern red cedar
*Thuja occidentalis* L.	Northern white cedar
*Thuja plicata* Donn ex D. Don	Western red cedar
*Widdringtonia* Endl. species	

**Table 2:** Wood essential oil compositions (percentages) of *Callitropsis nootkatensis* (D. Don) Oerst.

Compounds	Xiong 2000 ^a^	Liu 2009 ^b^	Khasawneh et al., 2011 ^c^	Commercial 2016 ^d^	Commercial 2020 ^d^
Carvacrol	27.2	12.5	35.4	7.3	9.1
Valencene	2.5	2.8	1.5	7.2	6.9
Nootkatene	13.3	17.3	20.1	48.4	46.9
Nootkatol	5.7	tr ^e^	5.2	nd ^f^	0.4
Valencen-13-ol	5.6	6.4	6.4	3.1	3.5
Nootkatone	12.9	9.7	17.4	3.0	4.6
Nootkatin	xtal ^g^	3.1	3.5	0.2	0.8

^a^ Yeping Xiong MS Thesis 2000 [18], ^b^ Xinfeng Liu MS Thesis 2009 [19], ^c^ Khasawneh et al., 2011 [20]. ^d^ Commercial essential oil sample from the collection of the Aromatic Plant Research Center (APRC), ^e^ tr = trace (< 0.05%), ^f^ nd = not detected. ^g^ Compound crystallized from the oil.

**Table 3:** Major components (percentages) reported in the wood essential oil of *Cedrela odorata* L.

Compounds	Brazil [27]	Nigeria [28]	Costa Rica [26]		Colombia [26]
α-Cubebene	8.0	nd	tr	tr	0.3
α-Copaene	15.6	0.7	0.3	0.2	1.3
α-Cedrene	nd	17.6	nd	nd	nd
β-Gurjunene (Calarene)	3.4	nd	5.5	5.7	nd
*trans*-α-Bergamotene	2.7	5.6	nd	nd	tr
α-Curcumene	nd	12.3 ^a^	nd	nd	nd
β-Curcumene	6.8	nd	13.0	12.8	2.2
δ-Cadinene	11.7	nd	26.0	26.3	53.2
(*E*)-Nerolidol	8.0	9.2 ^b^	1.2	1.1	nd
γ-Eudesmol	nd	8.8	nd	nd	0.1
α-Cadinol	1.5	nd	4.7	5.0	2.7
α-Eudesmol	nd	5.4 ^c^	nd	nd	nd
β-Bisabolol	0.5	11.0 ^d^	1.2	1.3	tr

nd = not detected. tr = trace. ^a^ Identification probably incorrect; reported RI = 1524, but RI from Adams [29] = 1479. ^b^ Reported as (*Z*)-nerolidol, but based on RI, this is most likely (*E*)-nerolidol. ^c^ Identification probably incorrect; reported RI = 1598, but RI from Adams [29] = 1652. ^d^ Identification probably incorrect; reported RI = 1619, but RI from Adams [29] = 1674.

**Table 4:** Major wood essential oil components (percentages) of *Cedrus atlantica* (Endl.) Manetti ex Carrière.

Source	Ref.	α- Himachalene	γ- Himachalene	β- Himachalene	(*E*)-γ- Atlantone	(*E*)-α- Atlantone	Others
Morocco	[39]	11.6	7.5	33.8	2.5	12.1	
Morocco	[36]	15.8	12.1	50.9	0.0	3.7	
Algeria	[31]	15.0	11.2	31.6	0.0	0.0	
Algeria	[40]	2.1	2.3	3.7	0.9	1.6	28.1% himachalol
Morocco	[41]	15.5	10.6	42.9	2.1	2.5	
Morocco	[35]	14.4	1.0	29.0	19.7	16.9	11.7% *iso*-cedranol
Morocco	[42]	12.2	8.5	27.7	0.0	0.0	9.4% himachalol
Morocco	[42]	16.7	11.3	44.2	0.0	0.0	4.5% 6-camphenol
Commercial	[37]	16.6	10.4	46.4	0.9	0.0	
Morocco	[32]	10.9	6.9	33.8	1.9	11.2	
Commercial	[43]	14.6	9.3	39.4	0.8	4.0	
Morocco	[43]	10.8	8.3	19.7	1.1	10.1	
Morocco	[44]	11.4	0.5	26.7	0.0	6.0	7.8% 6-camphenol
Corsica	[45]	0.9	0.9	1.7	0.0	0.3	55.0% α-pinene
Corsica	[45]	6.0	5.5	7.6	0.0	2.0	48.8% himachalol
Lebanon	[46]	8.0	14.0	7.0	1.5	0.0	46.3% himachalol
Lebanon	[46]	6.1	15.8	6.6	2.3	0.9	42.2% himachalol
Morocco	[33]	5.7	4.8	14.6	0.0	28.8	7.1% himachalol, 4.4% deodarone
Commercial	[47]	12.7	8.3	33.5	1.3	6.2	
Morocco	[34]	5.1	3.3	15.1	0.0	19.3	13.1% 8-cedren-13-ol
Romania	[38]	16.8	13.0	39.2	0.0	0.0	

**Table 5:** Major components (percentages) reported in the wood essential oil of *Cedrus deodara* (Roxb. ex D. Don) G. Don.

Compounds	Commercial (APRC Collection) ^a^	Chaudhary et al. [59]	Kala et al. [60]
α-Himachalene	13.6 ± 2.1	17.1	15.4
γ-Himachalene	8.7 ± 1.1	12.6	7.4
β-Himachalene	34.8 ± 3.8	38.8	19.9
(*Z*)-γ-Atlantone	4.0 ± 1.4	2.3	5.1
(*E*)-γ-Atlantone	4.7 ± 1.5	2.4	5.4
(*Z*)-α-Atlantone	2.5 ± 0.6	1.4	3.4
(*E*)-α-Atlantone	11.2 ± 3.1	8.6	14.0

^a^ Averages and standard deviations based on 43 essential oils.

**Table 6:** Major wood essential oil components (percentages) of *Cedrus libani* (L.) A. Rich.

Compounds	Geographical Location					
	Antalya, Türkiye [66]	Tarsus, Türkiye [66]	Fethiye, Türkiye [66]	Tanourine, Lebanon [46]	Tanourine, Lebanon [65]	Tanourine, Lebanon [64]
α-Himachalene	12.8	11.5	4.9	7.1	10.5	9.6
γ-Himachalene	7.6	6.8	4.4	7.0	9.1	5.9
β-Himachalene	38.2	34.3	8.1	11.9	21.9	19.0
6,7-Epoxyhimachalene	nd	nd	7.5	0.1	nd	1.3
Himachalol	1.2	8.8	19.7	43.1	22.5	7.0 ^a^
(*Z*)-γ-Atlantone	1.1	0.4	0.3	nd	1.7	4.4
(*E*)-γ-Atlantone	1.0	0.5	0.3	nd	1.7	3.6
(*Z*)-α-Atlantone	1.1	2.1	1.9	nd	2.1	4.6
(*E*)-α-Atlantone	7.8	14.8	19.7	0.9	0.8	19.3

nd = not detected. ^a^ Reported as α-acorenol, but probably himachalol.

**Table 7:** Major components (percentages) in the wood volatiles of *Chamaecyparis lawsoniana* (A. Murray bis) Parl.

Compounds	[68] ^a^	[16] ^b^	[69] ^c^
α-Pinene	6.5	nr	nr
Fenchone	4.7	nr	7.4
α-Fenchol	5.5	1.3	11.1
Camphor	5.9	nr	9.9
Borneol	nr	2.1	12.2
α-Terpineol	14.3	8.6	29.5
α-Muurolene	4.2	6.6	nr
δ-Cadinene	8.2	17.0	nr
τ-Muurolol	2.7	19.7	nr
α-Cadinol	5.3	21.4	nr

^a^ Commercial essential oils. ^b^ Ethyl acetate extract (not an essential oil). ^c^ Incomplete analysis (there are peaks corresponding to monoterpenes, but the concentrations were not reported; there are peaks corresponding to sesquiterpenoids, but the concentrations were not reported). nr = not reported.

**Table 8:** Major components (percentages) in the wood essential oils of *Cryptomeria japonica* (Thunb. ex L.) D. Don.

Compounds	Geographical Location											
	Kitayama, Japan [70]	Shimane, Japan [73]	Shimane, Japan [77]	Nara, Japan [73]	Kitayama, Japan [75]	Commercial, Japan [71]	Okayama, Japan [74]	Okayama, Japan [74]	Okayama, Japan [74]	Azores [78] ^a^	Azores [78] ^a^	Azores [79]
α-Cubebene	0.8	0.1	0.2	3.0	0.8	2.1	12.8	10.5	10.0	0.1–0.4	tr-0.4	0.2
*epi*-Cubebol	18.0	nd	nd	nd	16.4	nd	4.9	4.5	3.7	4.1–26.9	12.3–21.2	4.7
α-Muurolene	6.5	2.7	3.0	9.3	6.5	8.5	0.1	tr	0.1	2.0–3.3	1.5–2.9	0.5
Cubebol	16.4	nd	0.3	nd	18.0	nd	5.0	4.4	4.3	2.7–39.9	14.6–33.8	6.8
δ-Cadinene	21.4	13.9	16.8	25.9	21.4	22.8	tr	0.1	0.1	6.2–10.8	6.4–10.9	6.4
*trans*-Cadina-1,4-diene	nd	0.6	nd	nd	nd	nd	9.3	7.5	7.2	0.5–1.6	0.6–1.0	0.7
α-Elemol	nd	3.4	2.8	nd	1.5	6.7	0.1	0.6	0.4	1.7–14.1	1.8–9.2	2.6
1,10-di-*epi*-Cubenol	5.8	nd	nd	nd	5.8	nd	nd	nd	nd	5.5–17.2	4.0–18.4	0.1
1-*epi*-Cubenol	3.8	24.7	23.3	nd	3.8	7.9	6.3	4.5	4.4	nd	nd	10.7
Cubenol	nd	12.5	11.6	6.3	nd	4.6	4.9	3.3	3.5	nd	nd	nd
τ-Cadinol	nd	nd	nd	0.5	nd	1.8	nd	nd	nd	1.1–3.9	1.0–3.9	8.2
α-Muurolol (δ-Cadinol)	1.1	5.4	4.6	3.8	1.1	3.6	nd	nd	nd	nd	nd	4.3
α-Cadinol	nd	nd	nd	nd	nd	nd	1.1	nd	2.0	tr-5.2	tr-4.0	nd
β+α-Eudesmol	3.2	11.2	9.8	7.1	3.2	9.7	0.3	4.1	1.7	3.1–16.8	4.9–11.8	13.5
Abietadiene	1.0	0.3	0.4	nd	1.0	nd	13.7	8.2	9.8	nd	nd	0.5
Sandaracopimarinal	nd	1.0	1.7	nd	nd	nd	3.2	6.0	5.9	0.2–0.5	0.2–1.6	3.0
Sandaracopimarinol	nd	nd	nd	nd	nd	nd	13.7	17.1	17.6	tr-0.5	tr-2.0	5.5
*trans*-Ferruginol	nd	nd	nd	nd	nd	nd	11.3	10.6	10.2	tr-0.5	0.1–1.2	3.6

^a^ Ranges from 5 individual trees from two different populations from Faial Island. nd = not detected. tr = trace.

**Table 9:** Major components (percentages) in the wood essential oils of *Cupressus funebris* Endl.

Compounds	[83] ^a^	[81] ^b^				[81] ^c^	[82] ^d^	APRC ^e^	
α-Cedrene	26.4	3.4	1.8	0.7	2.2	22.2 ± 10.7	16.9	18.1	10.7
β-Cedrene	9.2	1.9	2.1	0.3	0.8	6.7 ± 2.3	5.7	5.0	3.2
*cis*-Thujopsene	29.9	2.3	0.8	1.4	18.7	27.4 ± 10.7	nd	29.6	10.5
Cuparene	3.4	nd	0.3	0.5	2.4	4.2 ± 2.4	5.4	4.4	29.4
Cedrol	9.6	43.9	54.6	72.8	45.0	13.8 ± 6.6	7.6	18.8	7.1

^a^ Source not indicated. ^b^ Pentane extracts of *C. funebris* wood samples. ^c^ Commercial “Chinese cedar wood oil” (ave. ± st. dev.). ^d^ Commercial essential oil. ^e^ Commercial essential oils from the Aromatic Plant Research Center (APRC) collection. nd = not detected.

**Table 10:** Major components (percentages) in the wood essential oils of *Juniperus ashei* J. Buchholz.

Compounds	[83] ^a^	[85] ^b^	[86] ^c^	APRC ^d^			
α-Cedrene	30.7	8.6	0.7	12.5	11.4	13.3	10.6
β-Cedrene	5.5	3.4	1.1	3.4	3.3	2.6	2.7
*cis*-Thujopsene	25.0	38.4	40.7	36.2	38.6	38.5	39.8
Cedrol	19.1	28.9	37.2	30.3	29.4	23.1	27.3

^a^ Source not indicated. ^b^ Commercial essential oil, “cedarwood oil from Texas” obtained from Kurt Kitzing GmbH, Wallerstein, Germany. ^c^ Supercritical carbon dioxide (SC-CO_2_) extract (not a true essential oil). ^d^ Commercial essential oils from the Aromatic Plant Research Center (APRC) collection.

**Table 11:** Major components (percentages) in the wood essential oils of *Thuja occidentalis* L.

Compounds	Weyerstahl et al. [97] ^a^	Anderson et al. [98] ^a^
Occidentalol	27.1	28.3
γ-Eudesmol	8.3	7.6
β-Eudesmol	9.1	9.1
α-Eudesmol	5.2	5.0
Occidenol	10.5	13.9
Occidol	21.2	21.3
Occidol isomer 1	5.3	5.1

^a^ Obtained from waste residues of cedar shingle mills in Quebec and New Brunswick (Canada) and Maine (USA).

**Table 12:** Major components (percentages) in the wood essential oils of *Widdringtonia* Endl. species.

Compounds	*W. nodiflora* [100]	*W. schwartzii* [100]	*W. nodiflora* [101] ^a^	*W. whytei* [102]
*cis*-Thujopsene	15.4	11.8	47.1	31.9
Germacrene D	3.6	18.3	nd	nd
*cis*-Thujopsadiene	nd	nd	1.8	7.1
Cuparene	3.0	14.3	4.0	3.8
Cedrol	10.4	10.1	10.7	13.6 ^b^
Widdrol	4.3	3.2	8.5	4.9 ^c^
τ-Cadinol	1.3	5.9	nd	nd
α-Muurolol	nd	13.5	nd	nd
α-Cadinol	7.2	nd	nd	nd
Thujopsenal (=Widdrenal)	1.8	11.0	3.2	nd

^a^ Identified as *W. cedarbergensis*. ^b^ The peak includes cedrol + β-himachalene + 3-thujopsanone. ^c^ The peak includes widdrol + 3-*iso*-thujopsanone. nd = not detected.

## Data Availability

All data will be made available upon reasonable request.
